# Study of Brain Circadian Rhythms in Patients with Chronic Disorders of Consciousness and Healthy Individuals Using Microwave Radiometry

**DOI:** 10.3390/diagnostics12081777

**Published:** 2022-07-22

**Authors:** Oleg A. Shevelev, Marina V. Petrova, Mikhail Yu. Yuriev, Elias M. Mengistu, Inna Z. Kostenkova, Maria A. Zhdanova, Sergey G. Vesnin, Igor Goryanin

**Affiliations:** 1 Federal Research and Clinical Centre for Resuscitation and Rehabilitology, 107031 Moscow, Russia; shevelev_o@mail.ru (O.A.S.); mpetrova@fnkcrr.ru (M.V.P.); myurev@fnkcrr.ru (M.Y.Y.); drmengistu@mail.ru (E.M.M.); kostenkovaie@mail.ru (I.Z.K.); mchubarova@fnkcrr.ru (M.A.Z.); 2 Department of Anaesthesiology and Intensive Care, Department of General Pathology and Pathological Physiology, Peoples’ Friendship University of Russia, 117198 Moscow, Russia; 3 Medical Microwave Radiometry (MMWR) LTD, Edinburgh EH10 5LZ, UK; vesnin47@gmail.com; 4 School of Informatics, University of Edinburgh, Edinburgh EH8 9YL, UK; 5 Okinawa Institute Science and Technology, Onna 904-0495, Okinawa, Japan

**Keywords:** medical microwave radio thermometry, brain, circadian rhythms, chronic impairment of consciousness

## Abstract

The study of circadian rhythms in the human body using temperature measurements is the most informative way to assess the viability of the body’s rhythm-organizing systems. Pathological processes can affect circadian rhythm dynamics in damaged organs. Severe brain damage that caused the development of disorders of consciousness (DOC) (strokes, traumatic brain injury) disrupts the activity of central oscillators, by directly damaging or destroying the periphery links, and the level of preservation of circadian rhythms and the dynamics of their recovery can be informative diagnostic criteria for patient’s condition assessment. This study examined 23 patients with DOC by using a non-invasive method for obtaining body and cerebral cortex temperature to compare with healthy controls. Measurements were made with a 4 h interval for 52 h beginning at 08:00 on day 1 and ending at 08:00 on day 3. The profile of patients with DOC showed complete disruption compared to healthy controls with rhythmic patterns. The results indicate that the mechanisms for maintaining brain circadian rhythms are different from general homeostasis regulation of the body. Use of microwave radio thermometry for the identification of rehabilitation potential in patients with DOC is a promising area of investigation.

## 1. Introduction

The study of circadian rhythms in the human body using temperature measurements is the most informative way to assess the viability of the body’s rhythm-organizing systems. Disturbances in their activity may be associated with the influence of environmental factors and the development of pathology, with a functional disconnection between central and peripheral oscillators, which include organ and intracellular pacemakers. Intracellular oscillatory systems, being the most phylogenetically ancient, are genetically fixed, and proteins encoded by genes are involved in periodic changes in diverse cellular processes. A gene network of intracellular oscillators provides rhythms that are synchronized with the environment, and intracellular oscillators have plasticity to adapt to actual needs required for the whole body functioning and regulation of central oscillators. Being controlled by the organ pacemakers, cellular rhythms have their own characteristics due to the actual needs and activity of internal oscillators [1]. Pathological processes can modify or suppress intracellular oscillators and disrupt the rhythmic homeostasis of tissue or the entire organ. Such malfunctioning may not be reflected using global indicators such as circadian changes of body temperature, sleep cycles, the activity of the cardiovascular and digestion systems, etc. [2]. This circumstance emphasizes that the oscillatory organization of the human body is normally heterogeneous, and that in pathology, the functional heterogeneity of rhythms can increase, being not only an object, but involved in the development of the disease [3]. The pathological process can affect the dynamics of circadian rhythms in the damaged organs [4].

There is evidence that the neoplastic process has a focal impact on tissue temperature in the tumor area, since the rhythms associated with malignant cell proliferation are largely non-circadian. In malignant cells, apparently, their own rhythmic signals are generated, which differ from healthy cells, and the degree of difference between local rhythms and the rhythm of the whole organism may be a reflection of the degree of development of the tumor. The measurements of such rhythms could be used for early diagnostics and more effective cancer treatment [4,5].

Brain damage can lead to disturbances in cerebral circadian rhythms, which was demonstrated using temperature sensors implanted in the brain parenchyma. A better outcome was shown in patients with preserved circadian rhythm and higher mesor (mean level) values, [6,7] which coincides with another study with similar results in half of the patients after severe traumatic brain injury (TBI) with maintained circadian rhythm of cerebral and body temperature [8,9]. These studies emphasize the importance of circadian rhythms, the presence of which reflects the higher potential of one’s own reparative responses.

The tumor process and severe brain damage have different mechanisms of circadian rhythm disturbances, affecting intracellular oscillators in the first case, and central oscillators in the second, destroying the circadian rhythms in the pathological brain [10,11]. The study of circadian rhythms in the brain could be useful for predicting the course and outcomes of the disease, including cerebral accidents.

Significant improvements in modern intensive care and resuscitation have led to a significant increase in the number of patients with severe brain lesions; some of them overpass coma and achieve a state of chronic disorders of consciousness (DOC)—a vegetative state (VS) and a state of minimal consciousness (MCS) for an indefinite period. VS is characterized by the presence of periods of wakefulness (eyes are open, gaze is not fixed) and periods of sleep with complete or almost complete absence of signs of purposeful behavior, awareness of oneself and the environment (awake unconsciousness). The period from the moment of development of impaired consciousness is, as a rule, at least 28–30 days after a cerebral accident [5]. In a state of MCS, there are distinct, although minimal, unstable signs of purposeful behavior, indicating the patient’s awareness of their own personality or the surrounding reality. They may not appear constantly, but are reproducible.

The amount of brain damage may not correlate with the level of consciousness retention, or disease outcomes [12].

The mere fact of alternating periods of sleep and wakefulness in VS patients is not evidence of the presence of circadian rhythm and remains a debatable issue, including in relation to MCS patients. Severe brain damage that caused the development of DOC (strokes, TBI, anoxic lesions) cannot but disrupt the activity of central oscillators, directly damaging them or destroying links with the periphery, and the level of preservation of circadian rhythms and the dynamics of their recovery can be an informative diagnostic criterion for assessing the patient’s condition [13,14].

Generally accepted recommendations for the diagnosis and prognosis of disease outcomes, approaches to therapy and rehabilitation of this category of patients have not yet been developed. There is no sufficiently deep understanding of the mechanisms of the pathogenesis of depression of consciousness in brain lesions and the processes of its recovery. An upward transition from VS and MCS to a higher level of consciousness is possible, but predictors of positive or negative dynamics of the disease development have not yet been found [15]. In this regard, it seems promising to study the circadian rhythms of the whole body and cerebral circadian rhythms in patients with DOC.

The temperature homeostasis of the body is characterized by a very high heterogeneity inherent in various parts of the body, individual organs, and to the greatest extent—the brain, where temperature differences between areas involved in excitation and in relative rest can reach several degrees [16]. The circadian rhythms of the whole body and cerebral circadian rhythms, despite the presence of temperature heterogeneity, are synchronized [17]. However, the mechanisms geared towards maintaining circadian homeostasis in various organs have their own specific features that can affect the indicators of rhythmic activity. In this regard, the brain is unique due to the highest thermal productivity and the peculiarities of the regulation of regional blood flow, which significantly distinguishes its ability to maintain circadian homeostasis of other organs and the whole body [18].

In particular, the metabolic activity of the brain ensures the release of about 20% of the heat of the whole body during normal conscious wakefulness whereas a brain mass is less than 2% of body mass [19]. Heat easily accumulates in the brain, which is facilitated by the position in the almost spherical shaped cranial cavity, and the peculiarities of the regulation of cerebral circulation. The main way to remove excess heat is the convection path, provided with a powerful influx of arterial blood (about 20% of the minute blood volume (MBV)). Due to the mechanisms of autoregulation, cerebral blood flow is relatively independent of the general hemodynamic within the known limits of changes in systemic arterial pressure [20]. This relative independence of the regulation of blood circulation in the brain regions and general cerebral perfusion can affect the processes of heat elimination and creates the basis for the relative independence of variations in cerebral and basal temperature. An increase or decrease in temperature in different parts of the brain may not be reflected in changes in body temperature, and it is not possible to judge the actual values of cerebral temperature according to measurements in the axial region or body cavities with sufficient accuracy [21]. At the same time, technologies for measuring cerebral temperature are now becoming a valuable tool for diagnosing and predicting the severity of the course and the outcomes of diseases caused by various brain lesions [22].

Invasive recording of brain temperature with implantable thermal sensors can be used to a limited extent in neurosurgical patients. For non-invasive registration, Magnetic resonance imaging (MRI) spectroscopy is used, which makes it possible to calculate the temperature values in various areas in the brain volume by calculation [23,24]. However, this technology is expensive, time consuming and not suitable for dynamic monitoring. Cerebral circadian rhythms studies require a non-invasive technique that allows multiple measurements during the day with sufficient accuracy. These requirements are met by microwave radio thermometry (MWR).

MWR is based on recording the power of the intrinsic electromagnetic radiation of tissues in the microwave range (1–7 GHz) [25]. This technology was developed to record the internal and skin temperature of tissues at normal state and disease, as well as for early diagnostics of different diseases including oncological [26] and nephrological illnesses [27]. It turned out to be applicable to assessing the internal temperature of the brain. Measurement accuracy by MWR is ±0.23 °C, which is similar to the results obtained when measuring temperature with sensors implanted to a depth of 3–4 cm in the brain parenchyma [28]. 

The circadian rhythms in humans and animals have been studied [29] in detail using various contact thermal and infrared sensors, however, the study of diurnal variations in brain temperature in humans using MWR has not been previously carried out.

The aim of the work is to study the cerebral circadian rhythms in normal individuals and patients with chronic impairment of consciousness by using MWR.

## 2. Results

### 2.1. Healthy Subjects

In healthy people, circadian rhythms had a distinct diurnal dynamic. In LBT and RBT, the maximum temperature values were registered at 16:00, amounting to 36.8 ± 0.14 °C (on the first day) and 36.8 ± 0.13 °C (on the second day) on the left, and on the right, 36.9 ± 0.14 °C and 36.9 ± 0.13 °C, respectively. The minimum values of the temperature of the cerebral cortex were recorded at 04:00 on the first and second days of the research, amounting to 35.7 ± 0.13 °C and 35.6 ± 0.11 °C, respectively, on the left, and 35.7 ± 0.11 °C on the right and 35.5 ± 0.12 °C.

The BT circadian rhythms had the same trends as the brain temperature, having differences in frequency and amplitude: maximum on the first and second days at 16:00: 36.7 ± 0.04 °C and 36.9 ± 0.04 °C. The minimum temperature was recorded at 04:00. On the first day, BT decreased to 36.3 ± 0.07 °C on the second day to 36.2 ± 0.08 °C. The temperature mesor of the LBT was 36.3 ± 0.04 °C, the RBT temperature mesor was 36.2 ± 0.05 °C, and the BT mesor was 36.4 ± 0.02 °C. So, BT mesor was lower than the temperatures of the cerebral cortex by 0.2 °C.

The periods of one complete temperature fluctuation for LBT and RBT exactly coincided and strictly corresponded to the circadian rhythm (24 h) on the first and second days. During the acrophase period (04:00), the temperatures of the LBT, RBT, and BT did not differ statistically, whereas during the bathyphase period (04:00) on the first and second days, the temperature of the LBT and RBT turned out to be statistically significantly lower than the BT. The maximum amplitude of temperature deviations of the LBT on the first and second days was 1.1 °C and 1.2 °C, the RBT was 1.2 °C and 1.4 °C, and the BT was 0.4 °C and 0.7 °C.

The BT oscillation period turned out to be slightly shifted. On the first day at 08:00, the axial temperature was 36.5 ± 0.15 °C and reached the closest values by 12:00 the next day (36.5 ± 0.08 °C), and at 08:00 on the third day it was significantly lower, also registered at 08:00 at the beginning of the study (36.0 ± 0.13 °C).

To detect relationships between temperature rhythms, a correlation analysis was performed (Pearson correlation analysis), comparing the dynamics of temperature changes in the LBT and RBT, in the LBT and BT, in RBT and BT at each time point of the study. The statistical analysis revealed strong significant positive correlations in the dynamics of changes in the temperature of the LBT and BT (correlation coefficient (CC) −0.899). Relationships between temperature changes in LBT, RBT, and BT turned out to be weaker than interhemispheric relationships, demonstrating positive relationships of medium strength (CC 0.446 and 0.426, respectively).

The results of processing the data array of 20 subjects give an idea of the relationships in temperature changes in the LBT, RBT and BT of the entire cohort and reflect general trends, without considering the individual characteristics of the circadian rhythms of each subject. For a more detailed elucidation of the features of the relationship between the temperature of the cerebral cortex and BT, we carried out the correlation analysis concerning the dynamics of temperature changes in each subject over two days at each time point of the study, including 08:00 on the third day: LBT and RBT, LBT and BT, RBT and BT. The obtained data (Table 1) confirmed the found general patterns obtained earlier. The CC of temperature changes in the LBT and RBT in all subjects varied within 0.933–0.714, reflecting the presence of significant strong positive relationships between circadian rhythms in both hemispheres. The results of the analysis of the relationship between circadian rhythms of the LBT, RBT and BT in each subject turned out to be heterogeneous. CC of circadian rhythms in LBT and BT varied within 0.645–0.267, and CC in LBT and BT from 0.907 to 0.193. In one subject, the associations of LBT and RBT with BT were negative: −0.213 and −0.155, respectively. At the same time, no psychological, somatic, or situational factors that distinguished this subject from the general cohort were not identified.

### 2.2. Patients with DOC

**Figure 1:**
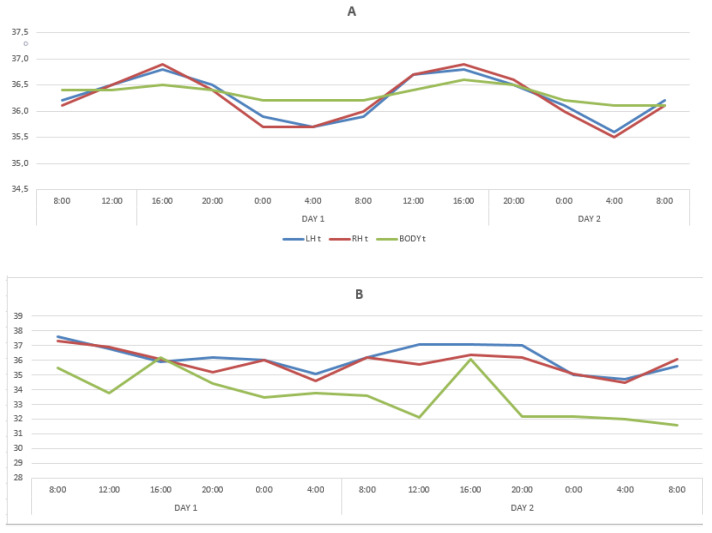
Changes in temperature of the LBT, RBT and BT in the cohorts of healthy subjects (**A**) and patients with DOC (**B**).

In patients with DOC, circadian rhythms variations in the LBT and RBT for two days were limited within the range of 35.8 °C–36.8 °C (amplitude 1 °C), without any noticeable periodicity. The maximum deviation was registered in the LBT and RBT at 20:00 on the second day (36.6 ± 0.06 °C), and the minimum in the LBT at 24:00 and 08:00 also on the second day. The BT minimum temperature values (36.0 ± 0.09 °C) were registered at 08:00 on the second day, and close to the minimum values at 12:00 and 04:00 on the second day and at 08:00 on the third. The maximum BT values were registered at 12.00 on the first day (36.08 ± 0.05 °C) and at 04:00 on the second day (36.08 ± 0.01 °C). These findings indicated that the circadian LBT, RBT, and BT rhythms were lost, which is illustrated by the data in Table 2 and the Figure 1B.

In the group of patients with DOC, strong significant positive correlations 0.717 were found in LBT and RBT. There were no correlations between LBT, RBT and BT during two days of the study. The CC values were 0.158 and 0.206, respectively.

A correlation analysis was carried out between the following combinations of variables LBT and RBT, LBT and BT, RBT and BT for each patient at every time point (Table 1), which revealed pronounced heterogeneity results and gave grounds for dividing patients into two groups.

The first group included eleven patients in whom interhemispheric connections were absent, weak, or moderate (CC < 0.6). In this group, the CC of LBT and BT turned out to be in the range of 0.153–0.581. 

Changes in the temperature of LBT and BT showed a spread in CC values from −0.524 to 0.439, and RBT and BT from −0.272 to 0.642, demonstrating the absence of inversion of connections, the absence or their moderate severity between daily changes in brain and body temperature.

**Figure 2:**
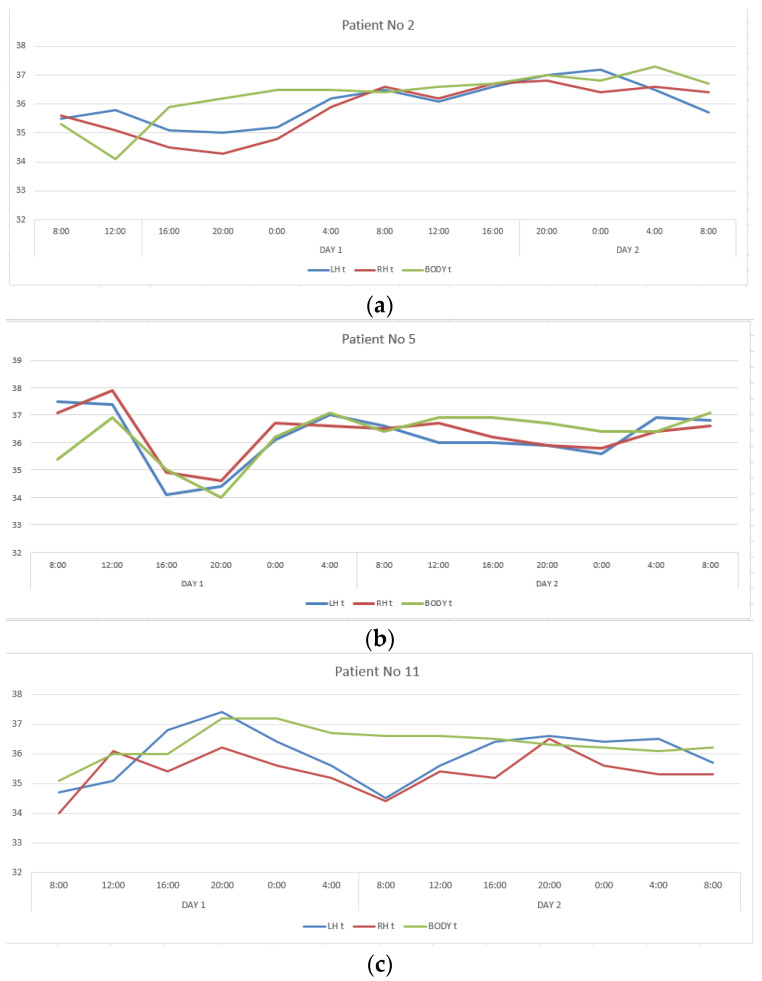

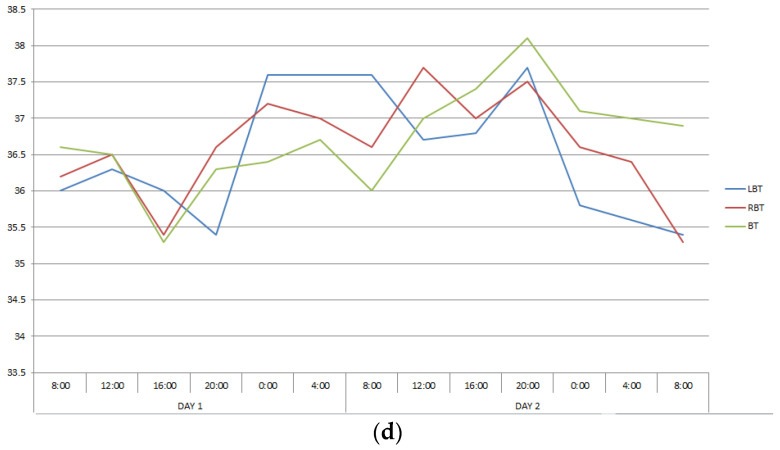
Changes in the LBT, RBT and BT in patients No. 2 (**a**), No. 5 (**b**), No. 11 (**c**), No. 17 (**d**) during the entire measurement period.

The second group included twelve patients in whom CC, reflecting interhemispheric connections, exceeded 0.6, ranging from 0.650 to 0.911. This group also turned out to be heterogeneous in terms of the severity of correlations between changes in the temperature of the cerebral cortex and body. In patients number two and five, CC describing the ratios of temperature variations of LBT and RBT, LBT and BT, RBT and BT corresponded to those in healthy people, and in patients number eleven and seventeen they were close to them (Table 1, Figure 2). In the remaining patients of this group, the correlations between brain and body temperature were negative or weak.

Despite the comparability of CC levels with healthy ones, there was no daily rhythm seen in changes in the temperature of the cerebral cortex and body in all patients with DOC (VS and MCS-minus).

Summary of results

According to the results of the conducted study, the circadian rhythms in healthy individuals were preserved and synchronized throughout the 52 h of measurement. However, the daily dynamics of temperature changes in the frontal cortex of the left and right hemispheres differed from the basal temperature variation. The periods of one complete temperature oscillation for both hemispheres exactly coincided and corresponded to the 24 h circadian rhythm, and the basal temperature was less distinctly synchronized with the circadian rhythm. It indicates that the brain circadian rhythms are autonomous from other body areas including the axillary region. Thus, the obtained results allow identification of certain differences in the circadian rhythms of brain temperature and basal temperature. The use of MWR is a more diagnostically valuable non-invasive method for studying circadian rhythms of the cerebral cortex temperature and facilitates detection of hidden circadian rhythms disruption that may not be reflected by measuring basal temperature. At the same time, in patients with DOC, regardless of age, gender and etiology of brain damage, a complete absence of circadian rhythm of body temperature and brain temperature was revealed.

The use of MWR made it possible to obtain data on the daily dynamics of changes in body temperature (BT) and temperature in left (LBT) and right (RBT) hemispheres of the cortex in the frontal regions, which differed significantly in healthy people and patients with DOC. The results obtained are shown in Figure 1 and in Table 2.

## 3. Discussion

The results obtained in the study of healthy people revealed distinct diurnal rhythms of temperature changes in the frontal regions of the cerebral cortex. It was found that the mechanisms for cerebral circadian rhythms are different from regulation of the general whole body circadian rhythms. The close relationship between changes in LBT and RBT could be explained by the equal influence of the central cerebral oscillators on both hemispheres and the presence of the liquid dynamic factor, which provides optimal conditions for heat transfer from the brain surface. Variations in LBT and RBT in healthy individuals are characterized by a distinct expression of circadian rhythms with a greater amplitude than changes in BT, the phases of which can shift relative to temperature rhythms in the frontal areas of the cerebral cortex, coinciding in periods of acrophase (time of the daily peak) and bathyphase (time of the daily minimum). The identification of these regularities shows that MWR could be useful for chronobiology studies.

During the bathyphase at night, the temperature of the cerebral cortex significantly decreases and is below body temperature. This observation seems to be very important because during the period of even such moderate hypothermia of the brain, endogenous neuroprotection reactions can develop, contributing to the restoration of the functions of neuron membranes during sleep, and disturbances in circadian cycles can accompany disorders in brain activity or even cause them [30].

Extensive brain damage that causes the development of DOC leads to disruption of circadian rhythms, apparently due to damage of the central cerebral oscillators or disruption of their links with peripheral pacemakers. It was not possible to reveal the degree of influence of impaired consciousness (VS or MCS) with the features of rhythm disturbances and individual characteristics of the correlations between changes in the temperature of the cerebral cortex and body in a relatively small cohort of patients. It can be assumed that with a positive course of the disease, circadian rhythms will be restored during the rehabilitation period. The in-phase temperature variations of the LBT and RBT, which do not have a circadian rhythm, are apparently explained by effective interhemispheric heat transfer due to the liquid dynamic component, which equalizes the absolute temperature values. There were no correlations between body temperature and the cerebral cortex in patients with DOC, emphasizing the relative independence of brain thermoregulation with basic circadian homeostasis.

Undoubtedly, the conducted pilot study requires a larger-scale trial, with more relevant control groups chosen according to similar age and etiology of the brain damage, since the possibility of age-related changes in circadian rhythms might be present [31]. 

However, the most important task in this study was to demonstrate the use of a new non-invasive method for registration of the circadian brain temperature rhythms, and to find out one of the main causes and/or consequences of persistent disturbances of consciousness in patients with severe cerebral injuries. For this reason, we enrolled relatively young volunteers in a control group, so that any age-related errors would be minimized. 

The results definitely demonstrate unaltered rhythms of the body and the brain temperature in healthy people, along with severe disruption of all the rhythms in every patient with DOC, regardless of age difference (the youngest patient included in the study was 24 years old, the oldest was 95 years old) nor the etiology of the cerebral injury (stroke/traumatic brain injury), confirming the existence of a relationship between an impaired consciousness and disruption of the circadian rhythm. We were able to obtain the data using radio thermometry and the traditional method of body temperature registration. Nevertheless, according to the obtained data, it appears that MWR is a more sensitive technique, than the conventional one and makes it a more significant and promising methodology in chronobiologic study.

In addition, considering the supposed positive effect of the night period of the bathyphase on the processes of neuroprotection and restoration of lost neuronal interconnections, it may be promising to use chronotherapy methods as part of rehabilitation measures by “imposing” rhythms of lowering the temperature of the cerebral cortex at night using craniocerebral hypothermia.

## 4. Materials and Methods

All procedures were approved by the Ethics Committee of the Federal Research and Clinical Centre for Resuscitation and Rehabilitology, Moscow, Russian Federation under Protocol No MO 01/18.

All participants or their authorized representatives signed a voluntary informed consent to participate in a clinical trial after explaining the objectives and algorithm of this study, as well as awareness of the on-going clinical methods conducted within the framework of this study.

The study included twenty healthy individuals (mean age was twenty-nine years, men—nine, women—eleven) and twenty-three patients with DOC, assessed by CRS-R (Coma Recovery Scale-revised) score less than eight, after severe brain damage (stroke -fifteen patients, TBI—eight patients), whose post-coma was characterized by access to the VS and MCS-minus over a period of 30–40 days (mean age was fifty-six years, men—fourteen, women—nine, patients in the VS—six, patients in the MCS—seventeen) Table 3.

In healthy people and patients with DOC, the internal and skin temperature measurements of the frontal areas of the cortex of the left (LBT) and right hemispheres (RBT) were taken every four hours, starting at 08:00 on the first day for two consecutive days. The study was completed by measurement at 08:00 on the third day. In the same period, the basal temperature in the armpit (body temperature—BT) was measured with a medical mercury thermometer in the axial region. Measurements were carried out under standard conditions at an ambient temperature of 23–24 °C and air humidity of 50–65%. To measure the skin and internal temperatures of the brain, the “RTM-01-RES” CE marked device (mmwr.co.uk) was used, which allows the user to measure the power of intrinsic electromagnetic radiation of the cerebral cortex in the frequency range of 3.2–3.7 GHz.

### Installation Procedure and Preparation for Measurement

Prior to measurement the device was turned on and warmed up for 30–40 min as recommended by the manufacturer, so that the antenna adapts to the environment and eliminates temperature or electromagnetic interference. Further, the antenna was tightly fitted to the palm skin at the base of the first finger of the operator. If the temperature readings were approximately 30–35 °C it was considered that the first check-up test was passed. The second test point was passed by firmly pressing the antenna to the right or left side of the neck at a height of 5 cm from the collarbone. The indicator should show a temperature of about 32–36 °C. After that, checking of the main set of the device was considered completed.

**Figure 3:**
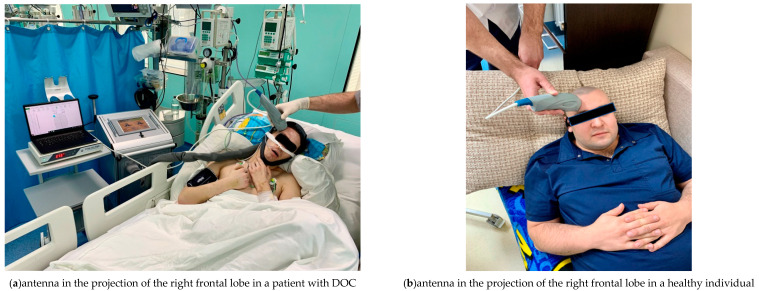
Using the MWR2020 (former RTM-01-RES) antenna in the projection of the left frontal lobe, the internal temperature of the cerebral cortex is being measured in patients with DOC (**a**) and healthy individuals (**b**).

For measurements, the MWR2020 antenna with a diameter of 32 mm was installed in the projection areas of the frontal lobes of the cerebral cortex on the left and right of the healthy individuals and patients with DOC (Figure 3), pressing it tightly to the skin surface. To obtain an accurate measurement of the internal temperature, it was necessary to tightly attach the working surface of the antenna to the examined area. The skin area had been prepared in advance so that there was no sweat or other possible fluids on it that might interfere with the measurement. A temperature was considered an artefact if was less than 32 °C or more than 42 °C. Artefactual readings were replaced by the average value of the three times remeasured temperature at the same projection area. The obtained temperature measurements were then entered into a spreadsheet in MS Excel (Microsoft Corporation. Microsoft Excel) and used for plotting as well as for database for statistical analysis. The results were obtained in August–September 2021.

Statistical data processing was carried out using the IBM SPSS Statistics for windows, version 21.0. Armonk, NY, USA: IBM Corp. Differences were considered significant at *p* < 0.05.

## Figures and Tables

**Table 1:** CC (correlation coefficient) of temperature changes in the LBT, RBT and BT during the entire period of measurements in healthy individuals and patients with DOC.

	Healthy Subjects			Patients with DOC		
№	CC LBT/RBT	CC LBT/BT	CC RBT/BT	CC LBT/RBT	CC LBT/BT	CC RBT/BT
1	0.788	0.469	0.595	0.224	−0.524	−0.004
2	0.724	0.645	0.907	0.870	0.547	0.459
3	0.881	0.552	0.528	0.731	−0.017	0.174
4	0.933	0.563	0.590	0.726	−0.079	−0.151
5	0.906	0.387	0.658	0.911	0.664	0.625
6	0.468	0.438	0.713	0.187	0.341	0.642
7	0.952	0.561	0.612	0.235	0.284	0.208
8	0.870	0.418	0.462	0.651	−0.114	−0.027
9	0.835	0.394	0.319	0.776	0.116	0.271
10	0.927	0.801	0.710	0.650	0.2019	0.005
11	0.718	0.411	0.573	0.662	0.439	0.435
12	0.915	0.436	0.372	0.742	0.351	0.129
13	0.854	0.405	0.575	0.766	−0.056	−0.272
14	0.869	0.437	0.630	0.391	0.382	0.555
15	0.842	0.267	0.277	0.581	−0.267	−0.145
16	0.709	0.677	0.428	0.720	0.178	0.089
17	0.933	0.331	0.304	0.655	0.535	0.180
18	0.896	−0.213	−0.155	0.309	0.398	0.069
19	0.910	0.405	0.315	0.153	0.044	−0.333
20	0.862	0.358	0.193	0.274	0.457	0.428
21				0.236	0.576	0.409
22				0.523	−0.187	0.213
23				0.523	0.421	0.397

**Table 2:** Mean temperature values of the cerebral cortex (LBT—left hemisphere and RBT—right hemisphere) and body temperature (BT) during 2 days in healthy people and patients with DOC.

	Healthy (20 Subjects)			Patients with DOC (23 Subjects)		
Time	t^0^		BT t^0^	t^0^		BT t^0^
	LBT	RBT		LBT	RBT	
08:00	36.2 ± 0.19	36.1 ± 0.18	36.5 ± 0.15	36.4 ± 0.1	36.3 ± 0.06	36.1 ± 0.05
12:00	36.5 ± 0.13	36.5 ± 0.14	36.6 ± 0.14	36.4 ± 0.04	36.3 ± 0.03	36.8 ± 0.05
16:00	36.8 ± 0.14	36.9 ± 0.14	36.7 ± 0.04	36.2 ± 0.05	36.1 ± 0.09	35.8 ± 0.07
20:00	36.5 ± 0.54	36.4 ± 0.13	36.6 ± 0.09	36.3 ± 0.06	36.2 ± 0.03	36.0 ± 0.09
24:00	35.9 ± 0.14	35.7 ± 0.14	36.3 ± 0.06	36.3 ± 0.03	36.4 ± 0.06	36.0 ± 0.06
04:00	35.7 ± 0.13	35.7 ± 0.11	36.3 ± 0.07	36.3 ± 0.08	36.3 ± 0.08	36.1 ± 0.05
08:00	35.9 ± 0.12	36.0 ± 0.12	36.2 ± 0.05	36.0 ± 0.02	36.0 ± 0.09	36.1 ± 0.04
12:00	36.7 ± 0.11	36.7 ± 0.14	36.5 ± 0.08	36.3 ± 0.04	36.4 ± 0.01	36.4 ± 0.03
16:00	36.8 ± 0.13	36.9 ± 0.13	36.9 ± 0.04	36.5 ± 0.09	36.5 ± 0.008	36.8 ± 0.01
20:00	36.5 ± 0.13	36.6 ± 0.15	36.6 ± 0.07	36.6 ± 0.06	36.6 ± 0.02	36.5 ± 0.03
24:00	36.1 ± 0.14	36.0 ± 0.13	36.4 ± 0.08	36.1 ± 0.06	36.1 ± 0.01	36.3 ± 0.09
04:00	35.6 ± 0.11	35.5 ± 0.12	36.2 ± 0.08	36.2 ± 0.06	36.1 ± 0.06	36.2 ± 0.09
08:00	36.2 ± 0.09	36.1 ± 0.12	36.0 ± 0.13	36.1 ± 0.07	36.1 ± 0.09	36.2 ± 0.02

**Table 3:** Study Sample Description.

	Healthy Individuals	Patients with DOC
**n**	20	23
**MEAN AGE**	29	56
**SEX**		
**MALE**	9	14
**FEMALE**	11	9
**CONSCIOUSNESS STATE**		
**VS**	0	6
**MCS**	0	17
